# Editorial for the Special Issue “Mathematical Modelling in Drug Delivery”

**DOI:** 10.3390/pharmaceutics17070871

**Published:** 2025-07-02

**Authors:** Fjóla Jónsdóttir

**Affiliations:** Faculty of Industrial Engineering, Mechanical Engineering and Computer Science, University of Iceland, Hjardarhaga 2–6, 107 Reykjavik, Iceland; fj@hi.is

This Special Issue highlights the essential role of mathematical modelling in pharmaceutical research, particularly in the development and optimization of drug delivery systems. Drug transport in biological environments is governed by multiple interdependent factors, including molecular diffusion, tissue permeability, and the physicochemical properties of drugs and formulations. In such systems, direct measurement is a key but often limited parameter. Mathematical modelling provides a framework that can systematically evaluate these complex processes.

This issue comprises five studies. One study examines oxaliplatin-based HIPEC using a computational model that incorporates changes in the tumour microenvironment due to bevacizumab, showing improved drug penetration with optimized treatment times [Contribution 1]. This work illustrates how simulation tools can be used to explore treatment strategies that would be difficult to evaluate experimentally. Another study applies finite element modelling to simulate methylene blue release from nanocellulose/nanoporous silicon composites, demonstrating how material composition affects diffusion dynamics [Contribution 2]. The findings demonstrate the potential of modelling to aid in the design of drug delivery materials where structural composition significantly affects performance. The third contribution proposes a mathematical model for drug dissolution from poly-disperse particles, accounting for the effects of solubility and wettability with minimal parameter fitting [Contribution 3]. This modelling approach can help guide early formulation choices where the rapid screening of candidate systems is needed.

The functionalization of breast implants with cyclodextrin polymer coatings is the subject of the fourth article [Contribution 4]. A unidirectional diffusion model, implemented in COMSOL Multiphysics software v. 6.0, is used to estimate key transport parameters, including diffusivity and partition coefficients. This study shows how modelling can be directly used to test release kinetics from implantable systems before physical prototypes are developed.

Finally, a comprehensive review paper [Contribution 5] categorizes mathematical models for oral drug absorption into three main types: data-driven, mechanistic, and first-principles-based models. The advantages and limitations of each are discussed, with examples given from the recent literature. This review also provides a primer for partial differential equations and their numerical treatment, helping bridge the gap between theory and practice for readers less familiar with mathematical methods.

Together, these contributions illustrate how modelling complements experimentation, offering predictive insights, supporting formulation design, and reducing development timelines. We thank the authors for their contributions and hope this collection encourages the wider adoption of mathematical modelling in pharmaceutical science.

